# Factors influencing cancer patients’ experiences of care in the USA, United Kingdom, and Canada: A systematic review

**DOI:** 10.1016/j.eclinm.2022.101405

**Published:** 2022-04-21

**Authors:** Saleh A. Alessy, Mohammed Alhajji, Janette Rawlinson, Matthew Baker, Elizabeth A. Davies

**Affiliations:** a Public Health Department, College of Health Sciences, Saudi Electronic University, Riyadh, Saudi Arabia; b Centre for Cancer, Society & Public Health, Comprehensive Cancer Centre, King’s College London, London, SE1 9RT, UK; c Behavioural Insights Unit (Nudge), Ministry of Health, Riyadh, Saudi Arabia; d Patient representative, National Cancer Research Institute (NCRI), Consumer forum, NCRI CSG (Lung) Subgroup, BTOG Steering Committee, NHSE CEG, UK; e Patient representative, National Cancer Research Institute (NCRI), Consumer Involvement Advisory Group, Consumer Forum, UK

**Keywords:** Patient experience, Cancer, Quality of care

## Abstract

The extent to which individual and structural factors influence cancer patients’ reports of their experiences are not yet well understood. We sought to identify which groups of patients consistently report poorer experiences and whether structural care factors might also be associated with better or worse reports. We conducted a systematic review of literature in PubMed and Web of Science with the date of last search as 27th of February 2022 following PRISMA guidelines. We focused on studies from three established population-based surveys datasets and instruments. After screening 303 references, 54 studies met the inclusion criteria. Overall, being from an ethnic minority group, having a more deprived socioeconomic status, poorer general or mental health status, being diagnosed with poor prognosis cancers, presenting to care through an emergency route, and having delayed treatment were consistently associated with poorer cancer care experiences. Conversely being diagnosed with earlier stage disease, perceiving communication as effective, positive patient-provider relationships, and receiving treatment with respect were overall associated with better reports of cancer care experiences. Improvement efforts aimed at delivering better experiences of patient-centred care need to take account much more explicitly patients’ differing characteristics, prognoses, and trajectories they take through their care journeys.

**Research in context**

**Evidence before this study**

Patient experience is evolving as an indicator of cancer care quality, and evidence suggests that care experiences vary significantly. Our previous review, in which we used mesh terms “patient experience” and “cancer” and “survival” to identify all articles published from January 1998 until March 2018 in the Medline database, returned 16 eligible articles, and showed variations in cancer patients’ survival by their reported experiences of care. However, no systematic review has yet synthesized and documented factors influencing cancer patients’ experiences.

**Added value of this study**

Our systematic review showed that patients’ experiences across several countries are consistently influenced by their demographic characteristics, aspects of their prognosis and system organisation issues. The factor most associated with negative patient care experiences is being from an ethnic minority background.

**Implications of all available evidence**

Inequality exists in reported patients’ care experiences at an international level. Rather than designing improvement initiatives that take a “one size fits all” approach to care experiences, policy makers should consider patients’ characteristics, their prognoses, and the differing trajectories they take through their care journeys.

Alt-text: Unlabelled box

## Introduction

Cancer patient experience is now developing both as a measure of cancer care quality to guide service improvements and as a research field in its own right.^1^, ^2^, ^3^ Recently there has been an increase in research that attempts to explore variation in cancer patient experiences. Emerging research has documented variations in patients’ experiences by either one or a combination of their demographic and disease progression factors.^4^, ^5^, ^6^, ^7^, ^8^, ^9^, ^10^ The extent to which various factors influence cancer patients’ experiences of care in different populations has not been assessed systematically due to the very recent development of this field.

Well-developed and large-scale surveys on patients’ experiences with cancer care gather information that can be used to monitor and improve cancer care policies. Three cancer patient experience surveys have currently been validated and used at a national population level to assess patients’ experiences with cancer care. Those are: The English Cancer Patient Experience Survey (CPES) in England,^11^ The Consumer Assessment of Healthcare Providers and Systems (CAHPS) in USA,^12^ and The Ambulatory Oncology Patient Satisfaction Survey (AOPSS) in Canada.^13^^,^^14^

We therefore systematically reviewed the available literature to identify all studies that have been published using the CAHPS, CPES, and AOPSS instruments or datasets in order to assess individual and structural factors found to influence cancer patients’ experiences of care. The aim of our study was to synthesize the evidence to determine which factors appear to consistently affect cancer patients’ experiences in different societies, with the goal of influencing cancer policy and efforts to improve cancer patients’ experiences internationally.

## Methodology

### Search strategy

We searched PubMed and Web of Science databases, with no year restrictions, to identify all studies (English language only) that used CPES, or CAHPS (focusing only on cancer care), or AOPSS instruments or datasets, with the date of last search as 27th of February 2022. Additionally, we used Google Scholar's advanced search feature “with the exact phrase” to identify published conference proceedings or any other possible study published in English using these surveys that might be indexed in other databases other than PubMed or Web of Science. Moreover, we also considered further articles for review that were mentioned in: the scoping review about cancer experience with care,^1^ the popular systematic review on patients’ experiences,^15^ our previous systematic review^16^ and the reference lists of identified studies. Mesh terms and keywords used for searching the database are listed in (Table 1).

**Table 1:** Mesh terms used in PubMed and Web of Science to retrieve all studies that either published from CPES, CHAHPS, and AOPSS datasets or used these survey instruments.

Database	National Cancer Patient Experience Survey	Consumer Assessments of Healthcare Providers and Systems	Ambulatory Oncology Patient Satisfaction Survey
PubMed	“CPES” OR "National Cancer Patient Experience Survey" OR "Cancer Patient Experience Survey" AND (cancer)	“Consumer Assessments of Healthcare Providers and Systems” or “SEER-CAHPS” or “HCAHPS” AND (Cancer)	“Ambulatory Oncology Patient Satisfaction Survey” OR “AOPSS” AND (cancer)
Web of Science	(TI= ("CPES”) OR TI= ("National Cancer Patient Experience Survey”) OR TI= ("Cancer Patient Experience Survey”) AND TS=(cancer)) Databases= WOS, MEDLINE, SCIELO Timespan=All years Search language=English	AB= ("Consumer Assessments of Healthcare Providers and Systems”) OR AB= ("SEER- CAHPS”) OR AB=("CAHPS”) AND TS=(cancer)	AB= ("Ambulatory Oncology Patient Satisfaction Survey”) OR AB=("AOPSS”) AND TS=(cancer)

### Eligibility criteria

The goal of this study is to identify which factors have been associated with patients’ reports of their cancer care experiences. We therefore included all journal articles that identified factors related to variation or differences in cancer patients’ experiences. To expand the scope of this review, published conference proceedings in English with a sufficient description of study aims, included population, and statistical analyses were also considered. Several published conference proceedings were therefore also found duplicated as original journal articles with slightly different titles. Where the research findings were found to be duplicated, we kept only the articles published in journals. Studies of patient reported outcomes measures or quality of life were not included on the basis that they do not ask patients questions about their experiences, but rather about symptoms, activities, or care outcomes. In addition, we excluded studies that (1) focused on patient satisfaction, (2) did not assess patient experiences (3) compared hospital performance (4) assessed the accuracy of patient experience data rather than reported patients’ experiences or (5) were reliability studies.

## Review process and quality assessment

**Figure 1:**
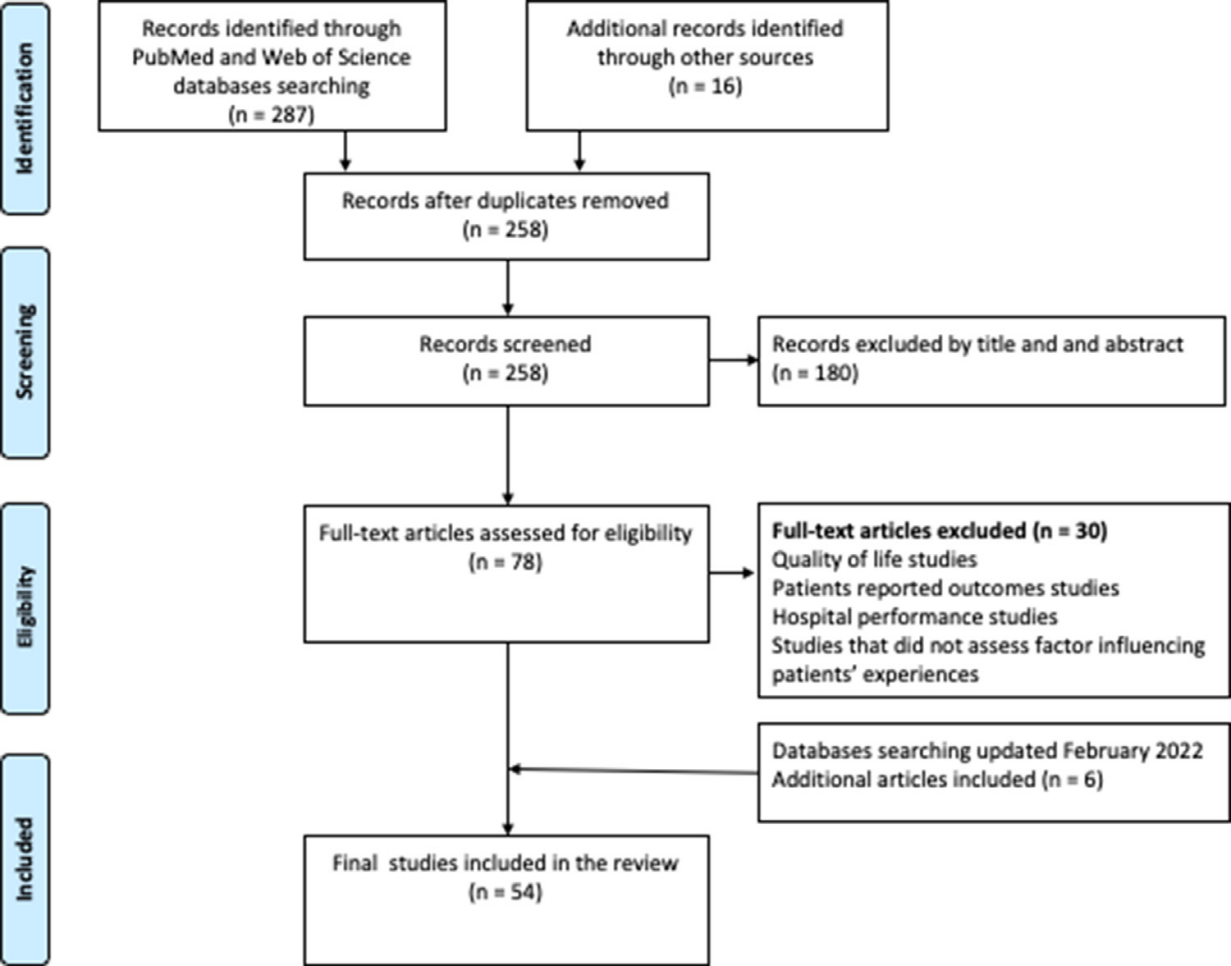
Flow chart of study inclusion process according to PRISMA guideline.

This study was conducted according to the Preferred Reporting Items for Systematic Reviews and Meta-Analyses (PRISMA).^17^ SA searched databases, initially screened articles, and chose the potential articles for a full text read. These potentially relevant studies were obtained as full text and assessed independently for inclusion by SA and ED, who then discussed and agreed on their inclusion. Finally, all study details were extracted from the full text publication by SA and then independently reviewed by MA and ED. Out of the 303 studies retrieved from our search, 54 studies met the inclusion criteria (Figure 1).

All quantitative full journal articles (*n* = 41) were then critically appraised and assigned a quality score using the Quality Assessment Tool for Observational Cohort and Cross-Sectional Studies designed by the National Institute of Health (NIH)^18^ (supplementary data). This step was carried out by an independent reviewer (MA) who was not involved in article selection to minimize the bias in assessing articles. The reason we chose this quality assessment tool was because most of the studies included in this review were cross sectional. Conference proceedings (*n* = 7) were not quality assessed as they were not full studies, and qualitative and mixed methods studies (*n* = 6) were not assessed due to heterogeneity in their designs. Most studies in this review followed the established process for the three major surveys with results and response rates being publicly reported. Studies were evaluated based on what was explicitly reported in the paper and we did not attempt to determine this by context. We did not exclude any articles based on their quality score, rather we aimed to assess the quality of the design and evidence in each study.

### Ethical approval

No ethical approval was required for this study.

### Role of the funding source

The funders of the study had no role in study design, data collection, data analysis, data interpretation, or writing of the report. SA and ED had access to the bibliographic search database. All authors had access to the included study data and all authors agreed with the final decision to submit for publication.

## Results

### Study characteristics

**Figure 2:**
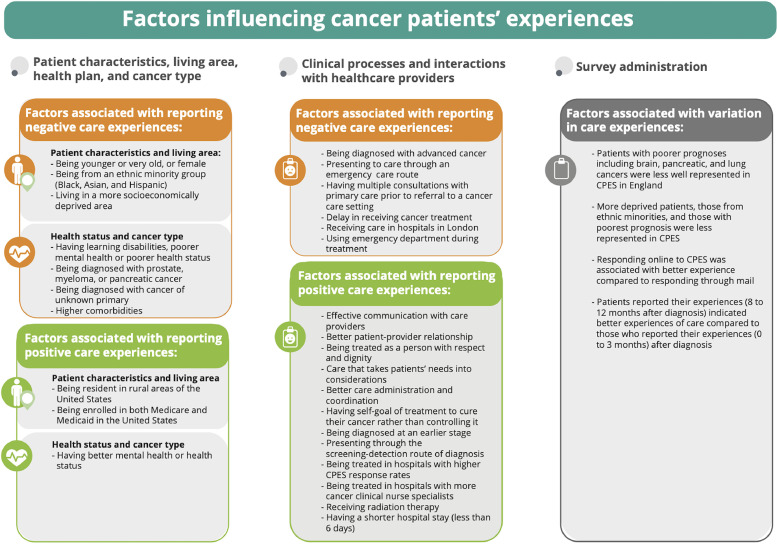
An evidence synthesis on factors affecting caner patients’ experience.

Most of the final included studies were from the United Kingdom due the availability of cancer patient experience datasets since 2010, with several recently published studies from Canada, USA, and Australia (Table 2). Authors’ names, the study aim, design, and a summary of findings were extracted for each study into Table 3. Most of the included studies were similar in design being quantitative cross-sectional studies including large numbers of patients diagnosed over one or several years (*n* = 48), but there was also one mixed method and five qualitative studies. Several factors were found to affect cancer patients’ experiences consistently across diverse cancer populations. These included patient demographic characteristics (age, sex, ethnicity, area of residence, socioeconomic status), health status, cancer type, cancer prognosis, patients’ presentation to cancer care, treatment facility location, and patients’ interactions with health providers. In addition, other structural factors such as health plan, hospital size, and survey responders’ characteristics were also found to influence patients’ experiences, but the extent of this varied between different populations. We summarized all factors influencing patients’ experiences into three main categories: (1) **patients’ cancer types and demographic characteristics**; (2) **patients’ interactions with the healthcare system**; and (3) **survey administration**. When studies crossed more than one category, we categorised them based on the main aim as stated in the study objective. Positive and negative influences on experiences, when these occurred in at least two or more studies are summarised as an evidence synthesis in Figure 2 and Table 4.

**Table 2:** Included Studies characteristics by design, type, country, category, and risk assessment.

Study characteristics	(*N* = 54)
**Design**	
Quantitative	48
Qualitative	5
Mixed methods	1
**Type**	
Journal article	47
Conference proceedings	7
**Country**	
United Kingdom	29
USA	20
Canada	4
Australia	1
**Category**	
Patients’ demographic and cancer type	30
Interactions with the healthcare system	19
Survey administration	5
**Risk of bias assessment overall score**	
Good	33
Fair	5
Poor	3
*Unassessed	13

⁎ This assessment only included 41 journal articles on cross sectional studies, and excluded 7 abstracts, 5 qualitative, and 1 mixed method study.

**Table 3:** Summary of included studies by design, aim, category of study and a short summary of the findings.

Authors	Title	Study design	Aim	Category	Summary
Lines et al. (A)., 2020^36^	Associations between perceptions of care experiences and receipt of mental health care among older adults with cancer.	A cross-sectional study using the SEER- CAHPS data for patients diagnosed with one from the 10 most prevalent solid tumour and completed CAHPS between 2006 and 2013	To examine whether older adults who received care for anxiety or mood disorders tend to report better care experiences.	1	Around 22% of the study sample (*n* = 4998) had both cancer and a claim for an anxiety or mood disorder. Worse mental health status was associated with worse care experience and was significantly associated with worse global care experience (0–10 scale driven from experiences with overall Care, personal doctor, and Specialist).
Chan et al., 2020^32^	Racial differences in lung cancer patient experiences with medical care and their association with cancer mortality: A SEER-CAHPS study	A cross-sectional study using the SEER- CAHPS data for 2603 lung cancer patients	To assess the association between a patient's race and their global ratings of care and composite scores (driven from three domains of patients’ experiences: centeredness, timeliness, and realized access)	1	This study showed variation in patients’ experiences with care by their race, and these variations were also associated with their subsequent mortality. Asians reported worse experiences with their ability to get care quickly, get needed drugs, get needed care, and rating of their overall health care compared to non-Hispanic white patients.
Farias et al., 2021^41^	Racial/ethnic Disparities in Patient Experiences with Health Care in Association with Earlier Stage at Colorectal Cancer Diagnosis	A cross-sectional study using the SEER- CAHPS date between 1997 and 2011 for colorectal cancer patients	To examine variations in patients’ experiences with patient-centeredness, timeliness, and realized access by race, and to determine the association between patients’ experiences with their stage at diagnosis.	1	This study showed that poor experiences with timeliness and realized access of care were more likely to be reported by patients diagnosed at advanced cancer stage. Asian patients reported worse experiences with getting care quickly and Black patients reported worse experiences for getting needed prescription drugs compared with non- Hispanic White patients.
Fauer et al. 2021^38^	Understanding quality and equity: patient experiences with care in older adults diagnosed with hematologic malignancies	A cross-sectional study using the SEER- CAHPS date between 2000 and 2015 for leukaemia or lymphoma cancer patients	To examine factors associated with patients’ experiences during the first year of their cancer diagnosis	1	Compared to patients form a white ethnic background, those from Asian, Indian Native, and Pacific backgrounds reported worse experiences of overall care. Multimorbidity was associated with better patients’ experiences with their personal doctor. Patients completing the survey 8 to 12 months after diagnosis reported a higher global rating of care compared to those who completed it 0 to 3 months after diagnosis.
Lyratzopoulos et al., 2012^19^	Variation in number of general practitioner consultations before hospital referral for cancer: findings from the 2010 National Cancer Patient Experience Survey in England	A cross-sectional study using 2010 CPES data in England for patients with 24 different cancers	To examine variation in patients’ experiences with the number of pre-referral consultations with a general practitioner (GP).	1	Three or more visits to the GP before hospital referral was considered a negative experience of care. The study found wide variation in patients’ experiences with GP visits by patients’ cancer types and demographic characteristics. Higher probability of three or more pre-referral consultations was found among young patients (aged 16–24 years), those from black ethnic minorities, women, and among patients with pancreatic, stomach, and lung cancers.
El Turabi et al., 2013^21^	Variation in reported experience of involvement in cancer treatment decision making evidence from the National Cancer Patient Experience Survey	A cross-sectional study using 2010 CPES data in England for patients with 38 primary cancers	To examine how experiences with involvement in decisions about treatment varied by patients’ characteristics and cancer type.	1	Patients’ experiences of involvement in decision making varied by their demographic characteristics and cancer types. Younger patients, those from ethnic minorities, and those with ovarian, myeloma, bladder and rectal cancers reported worse experiences compared with other patient groups.
Bone et al., 2014^20^	Inequalities in the care experiences of patients with cancer: analysis of data from the National Cancer Patient Experience Survey 2011–2012	A cross-sectional study using the 2012 CPES data in England for all cancer patients included	To explore inequalities in cancer patients' experiences by patient, clinical and trust-level factors.	1	This study showed inequalities in cancer patients’ experiences by their gender, age, ethnicity, and disability. After adjusting for patient, clinical and trust-level factors, female, non-white, younger patients, and patients with long-standing conditions (particularly those with learning disabilities or mental health conditions), were less likely to rate their overall care as excellent or very good
Saunders et al., 2014^7^	What explains worse patient experience in London? Evidence from secondary analysis of the Cancer Patient Experience Survey	A cross-sectional study using the 2012 CPES data and the Adult Inpatients survey in England for all cancer patients	To explore why cancer patients treated in London hospitals reported worse experiences of care compared with patients treated in all other English regions.	1	Patients with cancer treated by London hospitals reported worse care experiences. The differences were not explained by patient case-mix or whether the hospital was a teaching hospital. The study showed evidence of poorer experiences reported in London when comparing 10 of 16 experience aspects in both the CPES and the Adult Inpatients Surveys.
Saunders et al., 2015^22^	Inequalities in reported cancer patient experience by socio-demographic characteristics and cancer site: evidence from respondents to the English Cancer Patient Experience Survey	A cross-sectional study using all patients included in 2012 CPES data	To understand variation in cancer patients’ experiences among patients with different cancers.	1	Younger and very old patients, those from ethnic minorities, and women were more likely to report worse care experiences across CPES questions. Specifically, patients with multiple myeloma, ovarian, anal, hepatobiliary, and renal cancer reported notably worse experiences than patients with gynaecological, haematological, gastrointestinal, and urological malignancies respectively.
Stephens et al., 2015^23^	Keeping the customer satisfied #2 It Is OK to ask - who are we asking, and who participates? further findings from the National Cancer Patient Experience Survey 2013	A cross-sectional study using all patients included in the CPES 2013 data	To assess factors associated with variation in patients' experiences of being involved in cancer research.	1	There was little variation by gender in whether patients reported a conversation about taking part in research. There was a clear decline in being asked to participate in cancer research for patients aged over 75. Skin and urological cancer patients were less likely to be asked to participate in cancer research.
Mc Grath-Lone et al., 2015^24^	Exploring research participation among cancer patients: analysis of a national survey and an in-depth interview study	A mixed method study using the 2012–13 CPES data in England	To investigate variation in cancer patients’ experiences with being asked to participate in cancer research.	1	The study identified barriers to research participation at staff, patient, and trust level. Specifically, staff were less likely to discuss research with older patients. Asian and black patients were less likely to take part in research, while patients treated at specialist or teaching trusts had higher levels of discussion and participation in cancer research.
Lacey et al., 2016^45^	Presentations to general practice before a cancer diagnosis in Victoria: a cross-sectional survey	A cross-sectional study using CPES in care settings in Victoria, Australia for patients with 19 cancer types	To assess and understand variations in the number of general practitioner visits prior to a cancer diagnosis.	1	Patients with certain cancer types more frequently reported multiple GP visits, with 34% of all patients having visited a GP at least three times before being referred to hospital. Adjusting for age, sex, language, and socioeconomic deprivation, the highest number of GP visits were more likely to be made by patients with pancreatic, thyroid, vulvar, and multiple myeloma cancers, whereas the lowest number were by among patients with breast, cervical, and endometrial cancers.
Pinder et al., 2016^4^	Minority ethnicity patient satisfaction and experience: results of the National Cancer Patient Experience Survey in England	A cross-sectional study using the CPES data in England for all cancer patients included in the survey in 2012 and 2013	To explore reported experience of interacting with medical and nursing staff for cancer patients from ethnic minority backgrounds.	1	Patients from ethnic minority backgrounds reported lower satisfaction with and less positive experiences of care overall. Specifically, after adjusting for demographic factors, patients from ethnic minorities remained less positive in terms of having lower confidence in, and less understanding of healthcare professionals (including clinical nurse specialists, doctors, and ward nurses).
Shirk et al., 2016^30^	Patient experience and quality of urologic cancer surgery in US hospitals	A cross-sectional study using HCAHPS data from 2009 through 2011 for patients with genitourinary cancers	To determine whether there is an association between patients’ experiences and cancer surgical outcomes.	1	This study found a limited association between patients’ experiences and cancer surgical outcomes with variation by patient age, race, income, comorbidity, and cancer type. The study showed that patients’ experiences may be viewed as an independent quality domain rather than a mechanism by which to improve surgical outcomes.
Trenchard et al., 2014^25^	Ethnic variation in cancer patients' ratings of information provision, communication, and overall care	A cross-sectional study using the CPES data for all patients in England completing the survey in 2012	To examine variation in patients’ experiences with information provision and communication by their ethnic sub-categories.	1	Ethnic inequalities in cancer patients' experiences of information provision and communication were evident both between and within broad ethnic categories. Asian patients were less likely than White patients to receive an understandable explanation of treatment side effects. Specifically, Asian patients with Bangladeshi ethnicity were less likely to receive an understandable answer to their important questions.
Richard and Shaw, 2016^44^	How do myeloma patients' experiences before and during diagnosis compare to those for all cancers? Findings from secondary analysis of the National Cancer Patient Experience Survey 2014	A cross-sectional study using the CPES for all patients in England completing the survey in 2014	To examine whether myeloma patients reported worse experiences of diagnosis compared to all other cancer patients.	1	Myeloma patients reported worse experiences of diagnosis in the following care aspects: time prior to seeing a specialist, receiving information about diagnostic tests, and understanding at the time of diagnosis than the average for all other cancers. In addition, Myeloma patients reported longer times waiting for a diagnosis than the average for all other cancers and were less likely to feel that they received the information they needed about the tests and subsequent diagnosis.
Coronado et al., 2017^39^	The experience of patients with cancer during diagnosis and treatment planning: a descriptive study of Canadian survey results	A cross-sectional study using the AOPSS data in Canada for all cancer patients included in the survey between 2012 and 2016	To examine variation in patients’ experiences with patient-provider communication, during the diagnosis and treatment planning phases of cancer care	1	This study showed variation in patients’ experiences with patient-provider communication. Most respondents (92%) reported that their care provider told them of their cancer diagnosis in a sensitive manner and felt that they were provided with enough information about their cancer treatment. Across all cancers, prostate cancer patients reported the worst experiences with being referred for emotional support even though they reported having anxieties and fears upon diagnosis.
Cunningham and Wells, 2017^26^	Qualitative analysis of 6961 free-text comments from the first National Cancer Patient Experience Survey in Scotland	Qualitative analysis examining all cancer patients’ experiences reported in the first Scottish CPES in 2014	To understand patients' experiences of care, identify valued aspects and areas for improvement.	1	Although the majority of patients’ comments on their care were positive, there were a significant number of negative comments, especially about diagnosis care. The number of negative comments about care experiences varied by gender, age, employment status, and cancer type.
Halpern et al., 2017^31^	The health care experience of patients with cancer during the last year of life: Analysis of the SEER-CAHPS data set	Cross-sectional study using the SEER- CAHPS data between 1998 and 2011 for patients with all cancer types in the dataset	To assess factors influencing cancer patients’ experiences among individuals within one year before death.	1	Patients with higher general or mental health status were significantly more likely to report excellent experiences across all the measures examined. Sex, ethnicity, clinical characteristics, and education status were associated with patients’ experiences. Individuals in fee-for-service Medicare plans were more likely to report better experiences with health plans, getting care quickly, and getting needed care.
Hulbert-Williams et al., 2017^27^	The cancer care experiences of gay, lesbian and bisexual patients: A secondary analysis of data from the UK Cancer Patient Experience Survey	A cross-sectional study using the CPES data for all patients in England included in the 2013 survey	To assess whether there is variation in care experiences for patients based on their sexual orientation.	1	Around 0.8% of CPES responders in 2013 identified themselves as lesbian, gay or bisexual. After adjusting for age, gender and concurrent mental health comorbidity, less positive cancer experiences were reported by patients who identified themselves as lesbian, gay and (especially) bisexual.
Halpern, Urato et al., 2017^35^	Healthcare experience among older cancer survivors: Analysis of the SEER-CAHPS dataset	A cross-sectional study using the SEER- CAHPS data for breast, colorectal, lung, and prostate cancer patients completed CAHPS (1998 – 2011)	To examine experience of care among cancer patients and assess associations of patients’ characteristics with their experience.	1	Higher self-reported health status was associated with better experiences of cancer care with limited differences in patients’ experiences by sex or years since diagnosis. This association was significant for breast, colorectal, and prostate cancer patients. Better mental-health status was associated with better experience for lung cancer patients only. College-educated and Asian survivors reported lower care experiences.
Mollica et al., 2018^8^	Examining urban and rural differences in perceived timeliness of care among cancer patients: A SEER-CAHPS study	A cross-sectional study using the SEER-CAHPS (1998 - 2013) dataset for patients with breast, lung, colorectal, or prostate cancers	To examine whether there is variation in patients’ experiences by their place of residence at cancer diagnosis (urban vs rural).	1	Rural cancer patients were more likely to report better experiences with timely care than those in urban areas. Ethnic-minority rural patients were more likely to report negative experiences with accessing needed care as quickly. Black and Hispanic respondents from rural areas rated getting needed care lower than their counterparts residing in urban areas.
Lines et al., 2019^9^	Care experiences among dually enrolled older adults with cancer: SEER-CAHPS, 2005–2013	A cross-sectional study using the SEER- CAHPS date between 2005 and 2013	To understand the effects of poverty on self-reported care experiences among seniors diagnosed with cancer.	1	Patients who were enrolled in both Medicare and Medicaid tended to be poorer, have more functional and cognitive limitations, and have more medical needs compared with beneficiaries enrolled in Medicare or Medicaid alone. Cancer patients who were enrolled in both Medicare and Medicaid reported better care experiences with prescription drug plans and health plans than patients enrolled in Medicare-only.
Loiselle, 2019^33^	Cancer information-seeking preferences linked to distinct patient experiences and differential satisfaction with cancer care	A cross-sectional study using AOPSS data (2013–2017) for patients with 14 different cancers	To understand patients' needs and preferences as part of their care experiences.	1	60% of patients reported they wanted to actively seek information about their cancer, while around 40% did not want seek information about their cancer. Men were more likely to avoid asking about cancer information than women.
Farias et al., 2020^42^	Racial/ethnic differences in patient experiences with health care in association with earlier stage at breast cancer diagnosis: findings from the SEER-CAHPS data	Cross-sectional study using the SEER- CAHPS data for breast cancer patients who completed CAHPS survey between 1997 and 2011	To identify whether there is variation in patients’ experiences by their ethnicity and whether that is associated with stage at diagnosis.	1	Ethnic minorities reported poorer experiences with care preceding a diagnosis of breast cancer. In the adjusted analysis, black patients reported lower mean scores for getting care quickly, getting needed prescription drugs, getting needed care, and lower ratings of their overall health care compared to white patients. These worse experiences were more likely to be reported by patients with earlier stage breast cancer.
Mollica et al., 2020^34^	Perceptions of care coordination among older adult cancer survivors: A SEER-CAHPS study	A cross-sectional study using the SEER- CAHPS (2002 – 2013) dataset for over 14 different cancers patients	To examine sociodemographic and clinical variables associated with patients’ experiences with care coordination.	1	Several variables were associated with variation in patients' experiences with care coordination. Rural residence at diagnosis and visiting a personal doctor four times or more were associated with better care. in contrast, older age and seeing more specialists were associated with worse experiences with care coordination.
Wagland et al., 2017^46^	Differences in experiences of care between patients diagnosed with metastatic cancer of known and unknown Primaries: Mixed-Method Findings From the 2013 Cancer Patient Experience Survey in England	Qualitative analysis examining patients’ experiences reported in CPES 2013 for patients with cancer of unknown primary and those with metastatic disease of known primary in England	To explore differences in patients’ experiences between patients with cancer of unknown primary (CUP) and those with metastatic disease of known primary (non-CUP).	1	In a matched analysis for 2992 patients, there was a significant difference in care experiences between patients with cancer of unknown primary (CUP) and those with metastatic disease of known primary (non-CUP). CUP patients were more likely to report that they wanted more written information about their type of cancer and tests received, to receive their diagnosis from a GP and have seen allied health professionals, but less likely to have understood explanations of their condition or to have had surgery
Lines et al., 2020^37^	Illness burden is associated with care experience measures among Medicare beneficiaries surveyed before or after a cancer diagnosis	A cross-sectional study using the SEER- CAHPS date between 2007 and 2013 (no cancer type specified)	To understand patients ' Illness Burden Index (SCIBI) scores is associated with patients reported experiences.	1	In the adjusted analysis, the time between cancer diagnosis and survey response and SCIBI scores were associated with patients' care experiences. In particular, those with a higher illness burden reported worse care experiences with prescription drug plan customer service, doctor communication, and overall care.
Griffiths et al., 2013^52^	Is a larger specialist nurse workforce in cancer care associated with better patient experience? Cross-sectional study	A cross-sectional study using the 2010 CPES data for patients with 14 different cancers	To assess whether variation in the provision of cancer specialist nurses is associated with better reported patients’ experiences.	2	Cancer patients' experiences of care coordination and emotional support was better in trusts with more specialist nurses. Specifically, patients in these trusts reported better experiences with being treated and cared for well, being provided with enough emotional support, and being supported with the control of chemotherapy side effects.
Clucas, 2016^53^	Cancer patients' respect experiences in relation to perceived communication behaviours from hospital staff: analysis of the 2012–2013 National Cancer Patient Experience Survey	A cross-sectional study using CPES data in England for all cancer patients included in the 2012 survey	To explore whether communication behaviours from hospital staff are associated with cancer patients' experiences for being treated with respect.	2	Affective communication from hospital staff was associated with better patients’ experiences. Providing care with emotional support was associated with better reported experiences of being treated with respect although this varied by gender, ethnicity, age, comorbidity, treatment response, time since first treated, employment status, and type of cancer.
Mendonca et al., 2016^54^	Pre-referral general practitioner consultations and subsequent experience of cancer care: evidence from the English Cancer Patient Experience Survey	A cross-sectional study using CPES data for all patients in England completing the survey in 2012 and 2013	To examine whether visiting a GP (3+ visits) prior to diagnosis is associated with negative experiences of other cancer services using 12 CPES questions.	2	There was a negative association between multiple pre-diagnostic consultations with a GP and the experience of subsequent cancer care. Patients with 3+ pre-referral consultations reported worse care experiences in several CPES questions including involvement in the treatment decision, communication with clinical nurse specialist and being given information about treatment and care.
Saunders et al., 2016^55^	Do differential response rates to patient surveys between organizations lead to unfair performance comparisons? Evidence from the English Cancer Patient Experience Survey	A cross-sectional study using the CPES data for all cancer patients in England completing the survey in 2010	To explore possible associations between hospital-level survey response rates and patients’ experiences.	2	Hospitals that had higher CPES response rates also had more positive experience scores, which was partly explained by patient case-mix. In the multivariable analysis, associations between individual patient experience and hospital-level response rates were statistically significant in terms of managing late appointments, surgery admission delay, and providing correct documentation to patients.
Abel et al., 2017^56^	Emergency diagnosis of cancer and previous general practice consultations: insights from linked patient survey data	A cross-sectional study using the 2010 CPES data linked to route to diagnosis data for patients with the 18 most common cancers in England	To examine the experiences of patients who were diagnosed through emergency route with any previous or multiple primary care consultations.	2	Around a third of patients who were diagnosed through the emergency route never had any prior GP consultation. These patients were more likely to be male, older (≥85 years), living in deprived areas, and to be diagnosed with brain cancer. In addition, among emergency presenters with prior consultations, 41% reported 3+ GP visits, and these were more likely to be female, younger, and non-white and to be diagnosed with multiple myeloma.
Mollica et al., 2017^8^	Examining colorectal cancer survivors' surveillance patterns and experiences of care: a SEER-CAHPS study	A cross-sectional study using the SEER- CAHPS data for colorectal cancer patients diagnosed between 1999 and 2009	To examine the association between experiences of care and adherence to surveillance guidelines among Medicare Fee-For-Service beneficiaries.	2	Most of the 314 responders were highly satisfied with their care experience. This study showed that better experiences with patient-provider relationships improved adherence to office visits for colorectal cancer surveillance.
Salika et al., 2018^40^	Associations between diagnostic pathways and care experience in colorectal cancer: evidence from patient-reported data	A cross-sectional study using the 2010 CPES data inked to information on diagnostic route for colorectal cancer patients	To examine how different pathways to the diagnosis of colorectal cancer may be associated with patients’ experiences of care.	2	Screening-detected patients reported the best experiences of care, while emergency presenters reported the worst experiences. In 18 CPES questions about care experience, emergency presenters were more likely to report a negative experience for most questions, including those about diagnosis information and sufficient explanation before operations. Screen-detected patients were least likely to report negative experiences except for support from primary care.
Mollica et al., 2019^58^	Patient experiences of care in localized prostate cancer	A cross-sectional study using the SEER- CAHPS data between 1998 and 2011 for prostate cancer patients	To examine the association between treatment received (surgery, radiation, or no treatment) and patients overall experiences with care.	2	This study assessed patients’ experiences among localized prostate cancer patients (*n* = 507) receiving surgery, radiation, or no treatment. Respondents who received radiation were more likely to report their overall care as better than those not receiving this treatment. The overall care rating did not differ between patients who received surgery and patients received no treatment at all.
Pham, Gomez-Cano, et al., 2019^5^	Diagnostic route is associated with care satisfaction independently of tumour stage: Evidence from linked English Cancer Patient Experience Survey and cancer registration data	A cross-sectional study using the 2014 CPES data linked with data on diagnostic route and tumour stage at diagnosis for breast, prostate, colon, lung, and rectal cancers	To examine whether diagnostic route (e.g. screening or emergency presentation) is associated with cancer patients’ experiences independently of tumour stage.	2	Diagnostic route was associated with reported care experiences independently of cancer stage. In the adjusted analysis, emergency presenters had the highest likelihood of reporting a negative experience while screening-detected presenters had the lowest likelihood of reporting these experiences. Patients with advanced stage reported more negative experiences with little evidence of confounding between stage and diagnostic route, and no evidence for cancer-stage or cancer-route interactions.
Singer et al., 2019^59^	Patient Satisfaction After Lung Cancer Surgery: Do Clinical Outcomes Affect Hospital Consumer Assessment of Health Care Providers and Systems Scores?	A cross-sectional study using clinical data linked to HCAHPS survey data between 2014 and 2018 for lung cancer patients	To determine whether length of hospital stay affects HCAHPS scores.	2	Length of hospital stay (6+ days) after having lung resection for cancer was negatively associated with overall satisfaction scores (driven from several HCAHPS experience questions). In the adjustment analysis, increasing length of stay was associated with worse patient satisfaction in the aspects of communication with physicians and nurses (less likely to report that doctors gave understandable explanations and that nurses listened carefully).
Fitch et al., 2019^60^	Exploring the perspectives of patients about their care experience: identifying what patients perceive are important qualities in cancer care	Qualitative analysis examining cancer patients’ experiences reported in AOPSS in Canada between 2012 and 2016	To explore patients’ experiences of their care and identify aspects of care patients thought were important.	2	Out of 6232 patients’ comments, four themes were identified: characteristics of a "positive" experience, personal care, interaction with health care providers, and service delivery. Respondents reported that being treated as a person with respect and dignity, clear communication with staff, access to relevant and timely information, and care that takes their needs into account are important aspects of their experiences.
Gomez-Cano et al., 2019^6^	Patient experience drivers of overall satisfaction with care in cancer patients: Evidence from responders to the English Cancer Patient Experience Survey	A cross-sectional study using 2015 data CPES in England for all cancer patients included in CPES	To examine which aspects of care experience are the key drivers of overall satisfaction with cancer care.	2	Overall, out of 68,340 English patients who responded to CPES in 2015, 86% were highly satisfied with their cancer care. The strongest predictors of overall satisfaction with cancer care across all frameworks were responses to two questions on experience: the care administration and the coordination.
Watson er al., 2020^66^	A Cross-Sectional Analysis of Ambulatory Oncology Experience by Treatment Intent	A cross-sectional study using AOPSS data (2019) for patients with over 14 different cancers	To assess patients' perceived treatment goal, their experiences, and their satisfaction.	2	Out of 2104 participants, those who reported that their self-goal of treatment was to cure their cancer rather than controlling it reported better care experiences in five care aspects. These are access to care, coordination of care, respect for patient preferences, communication, and emotional support.
Fauer et al. ., 2020^61^	Health care experiences for older adults diagnosed with leukaemia and lymphoma: Factors associated with emergency department use, timeliness and access of health care	A cross-sectional study using the SEER- CAHPS date between 2002 and 2015 for leukaemia or lymphoma cancer patients	To examine the association between the emergency department, use in the first year of diagnosis and patients' experiences with care.	2	In the adjusted analysis, patients who had used an emergency department were more likely to report worse care experiences with getting care quickly and getting needed care aspects. The association was stronger for the “getting needed care”, and constant when comparing rural residents vs. urban.
Halpern et al., 2020^62^	Associations between shared care and patient experiences among older cancer survivors	A cross-sectional study using the SEER- CAHPS data for more than 8 cancer types patients completed CAHPS between 2000 and 2011.	To compare experiences of care between cancer patients who received shared care (a care pathway in USA), and those who did not.	2	Compared with other care pathways, shared care was not associated with better care experiences. This study concluded that patients receiving shared care reported similar care experiences to those receiving oncologists led care or primary care providers-led care.
Hamad et al., 2020^67^	Time-to-Treatment after Cancer Diagnosis and the Patient Experience with Care: A SEER-CAHPS Study	A cross-sectional study using the SEER- CAHPS (1998 – 2013) dataset for prostate, breast, lung, bladder or colorectal cancers	To assess if there is an association between time-to-treatment and cancer patients' experiences.	2	The adjusted multivariable analyses showed that delays in receiving cancer treatment were associated with reporting worse care experiences. Patients treated >6 months after their cancer diagnosis reported worse overall care, health plan, and getting needed care measures than patients treated less than two months from diagnosis.
Abel et al., 2016^47^	Post-sampling mortality and non-response patterns in the English Cancer Patient Experience Survey: Implications for epidemiological studies based on surveys of cancer patients	A cross-sectional study using the CPES data for all cancer patients in England included in the 2010 survey.	To assess if CPES sampling processes, post-sampling mortality and non-response can influence the CPES representativeness.	3	The overall response rate to CPES was 67%, being >70% for the most affluent patients and those diagnosed with colon or breast cancer, and <50% for Asian or Black patients. Patients with brain and pancreatic cancers had the highest risk of post-sampling mortality meaning that they had initially been included in the survey sampling but died and were removed before the survey was distributed.
Alessy et al., 2019^48^	How representative are colorectal, lung, breast and prostate cancer patients responding to the National Cancer Patient Experience Survey (CPES) of the cancer registry population in England? A population-based case control study	Population-based case-control study using 2010 - 2014 CPES data linked with cancer registration data in England for colorectal, lung, breast, and prostate cancers.	To assess the representativeness of CPES responders compared with the wider English cancer registry population In England.	3	CPES responders with colorectal, breast, lung and prostate cancers do not necessarily represent all patients with these cancers in terms of demographic characteristics and tumour stage at diagnosis. Across all cancer types, survey responders were younger, more likely to have a White ethnic background, to be resident in less deprived areas, and diagnosed with earlier stage disease although this varied between cancers. Survey responders also had higher median survival than the cancer registry population across all four cancers.
Pham, Abel, et al., 2019^50^	Predictors of Postal or Online Response Mode and Associations with Patient Experience and Satisfaction in the English Cancer Patient Experience Survey	A cross-sectional study using the 2015 CPES data for all cancer patients in the dataset.	To examine predictors of postal or online response mode, and associations with patient reported experience	3	Around 8% of CPES responders completed the survey online in 2015. Online and postal CPES responders tend to differ in their characteristics and rating of their care experiences. In the adjusted analysis, male, younger (<55 years), least deprived, and non-white patients were more likely to respond online. Patients responding online were more likely to report an overall satisfied experience of care.
Nartey et al., 2020^49^	Is the English Cancer Patient Experience Survey representative? A comparative analysis with the National Lung Cancer Audit	A cross-sectional study using CPES data from 2010 - 2015 in England for lung cancer patients.	To assess the representativeness of lung cancer patients responding to CPES in England.	3	There is a low representativeness of lung cancer patients who responded to CPES between 2010 and 2015 compared with the general lung cancer patients in England. Only 7% of all lung cancer patients were included in CPES. Older patients, those from more socioeconomically deprived areas, those with the worse performance status, multiple comorbidities, and patients diagnosed via emergency presentation were under-represented in CPES.
Alessy et al., 2021^64^	Being assigned a clinical nurse specialist is associated with better experiences of cancer care: English population-based study using the linked National Cancer Patient Experience Survey and Cancer Registration Dataset	Population-based cross- sectional study using 2010 - 2014 CPES data linked with cancer registration data in England for colorectal, lung, breast, and prostate cancers.	To assess whether being assigned a clinical nurse specialist is associated with better cancer patients' experiences	2	Patients who reported being given the name of a CNS in the CPES reported better care experiences on four aspects of care: involvement in treatment decisions, care coordination, treatment with respect and dignity, and overall care experience.
Dalby et al., 2021^51^	Analysis of local qualitative cancer patient experience alongside the 2019 results of the UK National Cancer Patient Experience Survey	A qualitative study using ten questions from the CPES in a local population	This study aimed to provide a in depth analysis of the results of the 2019 CPES.	3	The overall national CPES results do not always reflect focus group or interview findings using the same questions. CPES used on its own without qualitative data might not always be helpful for developing accurate improvement plans for cancer patient experiences.
Prue et al., 2021^65^	Exploring patient experiences of cancer care in Northern Ireland: A thematic analysis of free-text responses to the 2018 Northern Ireland Patient Experience Survey (NICPES)	A qualitative study using CPES data from 2018 in Northern Ireland	The study aimed to analyse free-text responses from CPES in Northern Ireland in 2018 to understand patients’ experiences of care	2	Overall, respondents reported very positive experiences of the cancer service in Northern Ireland. Positive experiences were attributed to the caring and professional nature of cancer staff, but some specific concerns about aftercare and perceived disconnect between primary and secondary care.
Nartey et al., 2022^28^	Using patient experiences to evaluate care and expectations in lung cancer: analysis of the English Cancer Patient Experience Survey linked with the national cancer registry	A cross-sectional study using CPES data from 2009 - 2015 in England for lung cancer patients	To assess lung cancer patients’ experiences of care according to demographic and clinical characteristics.	1	Patients’ experiences varied according to demographic and clinical characteristics. Patients aged between 65 and 80, those living in the most deprived areas, those who were diagnosed at lung cancer stage IIA–B, and those diagnosed through inpatient elective admissions were more likely to report positive care experiences.
Snyder et al., 2021^63^	Association of Patient Experience with Guideline-Concordant Colon Cancer Treatment in the Elderly	A cross-sectional study using the SEER- CAHPS data between 2003 and 2013 for colon cancer patients.	To assess whether there is an association between better cancer care experience and guideline-concordant care (GCC) delivery among elderly patients with colon cancer	2	Of elderly patients who received the Guideline Concordant Cancer Care pathway, those with stage III colon cancer reported better experiences with getting needed care.
Navarro et al., 2022^43^	Racial/ethnic disparities in patient experiences with care and Gleason score at diagnosis of prostate cancer: a SEER‑CAHPS study	A cross-sectional study using the SEER- CAHPS data between 1997 and 2011 for prostate cancer patients.	To assess whether racial/ethnic differences in patient experiences with care are associated with disparities in Gleason score at diagnosis.	1	Gleason score varied by reported patient experiences with care. Among non-Hispanic white and non-Hispanic black respondents, the ability to get needed prescription drugs was associated with lower odds of a higher Gleason score at diagnosis.

**Abbreviations:** CPES = Cancer Patient Experience Survey; CHAPS or HCHAPS = Hospital Consumer Assessment of Healthcare Providers and Systems; AOPSS = Ambulatory Oncology Patient Satisfaction Survey; GP= General practitioner. Category = 1) patients’ cancer types and demographic characteristics; 2) patients’ interactions with the healthcare system; and 3) survey administration.

**Table 4:** Number of studies by country and type of factors that have reported to be associated with cancer patients’ experiences of care.

	Country				
Factors associated with variations in cancer patients’ experiences*	Australia	Canada	United Kingdom	United States	Total
Cancer type or stage at diagnosis	1	1	2	1	5
Care coordination or access to care	–	1	8	8	17
Ethnicity, age, or other sociodemographic factors	–	1	12	7	20
Mental or overall health	–	1	–	4	5
Survey methods	–	–	5	–	5
Treatment location	–	–	2	–	2
**Total**	**1**	**4**	**29**	**20**	**54**

Some studies reported more than one factor; hence we categorized such study based on the first aim.

### Patients’ cancer types and demographic characteristics

A total of 30 studies linked variation in patients’ experiences to their demographic or disease characteristics. A full list of these studies’ citations and summaries can be found in Table 3. Published studies from several yearly rounds of CPES documented variation in patients’ experiences with primary care, involvement in treatment decisions, health provider communication, and overall care experience by their sex, age, and ethnicity.^4^^,^^19^, ^20^, ^21^, ^22^, ^23^, ^24^, ^25^, ^26^, ^27^, ^28^ These studies showed that overall, younger patients reported less positive experiences than older ones,^20^^,^^22^^,^^24^ females reported less positive experiences than males,^20^ and non-white patients reported less positive experiences than those from the white population.^4^^,^^20^^,^^22^^,^^29^ Similar variation in cancer patients’ experiences by their sex, age, and ethnicity were also found in studies from USA,^30^, ^31^, ^32^ and Canada.^33^

Furthermore, residence area and socioeconomic status were found to influence patients’ experiences. For example, patients from the urban capital city of London tend to report less positive experiences than those from other areas of England.^7^ In addition, cancer patients in rural areas in the United States report better experiences with timely care than those in urban areas,^8^^,^^34^ and patients who are enrolled in both Medicare and Medicaid (and who therefore tend to be more deprived) report better care experiences with prescription drug plans and health plans than patients enrolled in Medicare-only.^9^ Moreover, Halpern and colleagues assessed care experiences among patients with cancer in the year before death, and found better mental health and being a longer time before death were associated with better experiences.^31^ Another study using the same dataset found that better reported health status and better mental health were associated with reporting a better care experience, while worse mental health was associated with worse care experiences.^35^^,^^36^ Patients’ comorbidities were also shown to influence their experiences, with patients who had higher illness burden reporting worse care experiences, but the direction of this association varied between different cancer types.^37^^,^^38^

Variations in patients’ experiences of cancer care were not limited to patient sociodemographic characteristics but were also documented by cancer site and disease prognosis. For example, patients with prostate cancer tend to report the worst experiences of being referred for emotional support in Canada.^39^ In terms of experience with cancer diagnosis, patients who were diagnosed with late stage disease were more likely to report worse care experiences in England, ^40^and in the USA.^41^, ^42^, ^43^ Moreover, patients with multiple myeloma and pancreatic cancer were more likely to report negative experiences in England,^19^^,^^44^ while patients with thyroid, vulvar, and multiple myeloma cancers were more likely to report negative experiences in Australia.^45^ In addition, patients with cancer of unknown primary in England were more likely to prefer more written information about their type of cancer and tests received compared to those with metastatic disease of known primary.^46^ Generally, it seems therefore that patients presenting with uncommon cancers or more advanced disease, which means they are facing an uncertain prognosis, tend to report less positive care experiences.

### Survey administration

Five studies assessed how CPES sampling and the survey administration processes influenced reported patients’ experiences.^47^, ^48^, ^49^, ^50^ Abel and colleagues found that patients with brain and pancreatic cancers had the highest risk of post-sampling mortality meaning that individuals who had initially been included in CPES sampling, died before the survey mail out could be carried out and so were excluded.^47^ These findings about the limitations of CPES representativeness were also confirmed by two other studies that compared the survey responders to the general cancer population of the same cancer in England.^48^^,^^49^ These studies found patients who are included in CPES tend to be less deprived, more likely to be from a white background, and to have better prognoses.^48^^,^^49^ Survey methodology and the timing of response were also shown to influence patients’ reported experiences. Patients who responded online reported better overall experience of care than those who responded through mail.^50^ Also, patients who completed the survey between 8 and 12 months after their diagnosis reported a higher global rating of care compared to those who completed it between 0 and 3 months after.^38^ In addition, a qualitative study comparing the CPES 2019 results with those obtained using the same questions at focus groups and interviews suggested whether the survey questions were not specific enough to represent patients’ experiences in sufficient detail.^51^

### Interactions with the healthcare system

Nineteen studies linked variation in patients’ experiences to their interactions with the healthcare system or their care providers.^5^^,^^6^^,^^59^^,^^60^^,^^40^^,^^52^, ^53^, ^54^, ^55^, ^56^, ^57^, ^58^ First, patients’ presentation pathways to cancer care were associated with their reported care experiences.^5^^,^^40^^,^^54^^,^^56^ For example, studies using CPES datasets showed that patients who presented to cancer care through emergency care or those who had multiple consultations with primary care prior to referral tended to report negative care experiences with cancer care.^5^^,^^40^^,^^54^^,^^56^ This association was also shown in the USA where patients who had used an emergency department after their cancer diagnosis cancer reported worse care experiences.^61^ In contrast, patients reported better experiences when they were diagnosed at an earlier disease stage or presented to care through the screening-detection route of diagnosis.^5^^,^^40^ In the USA, no difference in care experiences was found for patients who received care through a shared care pathway compared to those receiving either oncologist led care or primary care provider-led care.^62^ However, another study from USA using the same dataset found that elderly patients with advanced stage colon cancer who received the Guideline Concordant Cancer Care pathway reported better care experiences with getting needed care.^63^

Care coordination and communication with healthcare providers were found to influence patients’ experiences. Specifically, studies from the UK, Canada, and the USA showed that patients reporting effective communication, better patient-provider relationship, being treated as a person with respect and dignity, care that takes patients’ needs into considerations, and better care administration and coordination^6^^,^^53^^,^^57^^,^^60^ as key aspects in improving their overall care experiences.^6^^,^^53^^,^^57^^,^^60^ In the UK, two studies of the role of clinical nurse specialists in cancer care found that contact with them was subsequently associated better experiences of involvement in treatment decisions, care co-ordination and being treated with more respect and dignity.^64^^,^^65^ Furthermore, better experiences in terms of the patient-provider relationship were also found to improve adherence to office visits for colorectal cancer surveillance in USA.^57^ In addition patients who reported that their self-goal of treatment was to cure their cancer rather than to control it reported better experiences with access to care, coordination of care, respect for patient preferences, communication, and emotional support.^66^ Patients’ experiences with care were also associated with hospital size and treatment received. For instance, patients reported better experiences in hospitals where there were more cancer specialist nurses.^52^ Additionally, patients who received radiation therapy were more likely to report their overall care as better than those not receiving this treatment.^58^ while those who had shorter hospital stays (less than 6 days) were more likely to report better experiences than those stayed longer.^59^ Delays in receiving cancer treatment were also associated with reporting worse care experiences. Patients in USA who were treated more than 6 months after their cancer diagnosis reported worse overall care, health plan, and getting needed care measures than patients treated less than two months from diagnosis. Finally, hospitals with higher CPES response rates also report better experiences. Since non-responders and late responders are less positive about their care, a low survey response is seen as an indicator of poorer experiences for a given hospital.^55^

## Discussion

Patient centred cancer care should be tailored to patients’ needs and preferences.^68^ This review finds that the patient experience research field is emerging in many countries^69^ and large amounts of data are now being collected and reported on patients’ experiences in many differing healthcare systems. Our study has revealed that a wide range of factors can influence cancer patients’ reported experiences including their demographic characteristics, health status, cancer type and prognosis, and interactions with particular aspects of the healthcare system, as well as the survey experience collection methods. Patients’ demographic and cancer characteristics were the factors associated in the most pronounced way with variation in patients’ experiences across all studies. Being from an ethnic minority group, having a more deprived socioeconomic status, or poorer general or mental health status, were factors consistently associated with poorer cancer care experiences. In addition, being diagnosed with advanced stage disease, less common cancer types that carried a poorer prognosis, presenting to care through an emergency route, and having delayed treatment were also associated with less positive experiences. Conversely being diagnosed with earlier stage disease, perceiving communication as effective, positive patient-provider relationships, and receiving treatment with respect and dignity were associated overall with better overall reports of cancer care experiences.

Several systematic reviews have previously focused on cancer patients’ experiences.^1^^,^^16^^,^^70^ Saunders and colleagues, for example, highlighted variation in the emerging patients’ experiences measures worldwide.^70^ These variations in care experiences by factors such as sex, higher comorbidity, and ethnic background are observed not only in the cancer patient experience literature, but are known to occur in patient experience results for many different health conditions.^71^

Our study summarised evidence gathered from specifically designed surveys on what factors impact patients’ experiences in three developed healthcare systems. In addition, Mollica and colleagues conducted a scoping review explaining the landscape of cancer patients’ experiences research in USA when introducing the SEER-CAHPS linked dataset. Our study examined subsequent evidence generated from recently published studies from SEER-CAHPS linked dataset, revealing variation in cancer patients’ experiences in USA. Although the structure of cancer care in USA is different from the UK and Canada, there were several consistent individual factors affecting patients’ experiences across these systems such as patients’ ethnicity and their cancer type.

To our knowledge, this is the first systematic review to focus on assessing factors influencing the experiences of cancer patients. In addition, this is the first such review to focus on studies that specifically aim to measure patients’ experiences and have been published from large patient experience datasets recently available in USA,^12^ England,^72^ and Canada.^13^ Based on our review design and inclusion criteria, the heterogeneity between studies was limited, and most showed a high degree of similarity in their purposes, design, sample size, and concepts. While this review is comprehensive in terms of searching several databases, studies that used other tools to assess patients’ experiences were excluded together with data from healthcare systems in other areas of the world. The review also did not include studies that focused on the relationship between cancer experiences and other cancer outcomes as other work has previously examined this.^16^ Some studies had more than one aim and while we assigned and categorised the first aim for this review, inevitably this led to some loss of information which future researchers might wish to avoid.

In addition, findings from this study may not necessarily represent all the included countries similarly. Although the included papers were from different countries, most of them were from the UK, followed by USA. This was mainly due to the nature of the existing literature in the field of cancer patient experience in these countries. Although the included studies have reported cancer care experiences for different ethnic groups, these studies were all from high income countries limiting the generalisability of this study to low resource and developing settings. In addition, previous research has also documented limitations in capturing the experiences of all cancer patient groups in patient experiences surveys, especially for ethnic minorities and lower survival groups.^47^, ^48^, ^49^

Findings from this study are important for policy makers, practitioners, and patient experience researchers as they emphasise that the experiences of patients with cancer are influenced by several different factors or a combination of them. Efforts to improve patients’ experiences or survivorship programs among individuals with cancer should carefully consider what might affect patients’ experiences to be able to prioritise or propose improvement initiatives. In addition, this study also shows that cancer care programs should not be designed as “one size fits all”. For example, several studies included in this review showed that there is a variation in patients’ experiences by cancer site with which patients present, even after adjustments for socio-demographic factors in both CPES and CAHPS. This raises the importance of more tailored experience improvement initiatives by oncology services and the need for programmes that take account of a range of patients’ characteristics, their different cancers, and the different trajectories they take through their care journeys. For example, patients in England who present to cancer care through emergency presentation tended to report poorer care experiences compared to patients presenting to care with screen-detected good prognosis cancer.^40^ This suggests that the speed and success of a relatively straightforward diagnostic phase can have far-reaching positive effects in terms of confidence that patients may feel in the cancer care system and its ability to care for them. Consequently, when a diagnosis has been difficult or delayed, more time and effort may be needed ‘to repair’ the effects of what appears an uncoordinated beginning to cancer care. In addition, the consistent difference in experiences reported by patients from non-white ethnic groups across three differing health systems suggests that more work is needed to understand what lies behind these experiences, how these may be improved and how to co-design solutions.

There is also emerging evidence that clinical nurse specialists play an important role in that their care is associated with better experiences of involvement in treatment decisions, care co-ordination and being treated with more respect and dignity.^64^ Further research on their role and other ways of co-ordinating care around individuals is needed. Specifically, our findings can also inform researchers and survey designers about the similarity and heterogeneity of patients’ experiences to inform new analyses about how to improve rather than to continue to simply document these differences. Our study has also documented the variety of instruments used to measure and analyse cancer patients’ experiences of care from one care setting to another, creating a synthesis of knowledge on how to approach cancer patients’ experiences reliably and effectively.^2^^,^^73^^,^^74^ These findings will also be of use to clinician and nursing leaders, managers, and policy makers charged with the responsibility of deciding just where to focus quality improvements efforts for individual hospitals, cancer centres, regional or indeed national populations. Understanding where the largest inequalities in experiences lie and the structural aspects of care that may underlie them makes it easier to decide where to concentrate efforts and maintain momentum. Finally, the coronavirus disease (COVID-19) pandemic has affected the capacity of cancer care in many countries causing services disruptions and leading to the implementation of telemedicine in cancer care.^75^ Patients’ experiences with the remote consultation and support will be even more vital to understand, given the digital divides worldwide.^76^ Further research both on the effect of the pandemic on care experiences and on effective ways to improve and sustain experiences using survey data and other qualitative data are urgently needed.

Patient experience is developing both as a quality indicator and as a research topic in many care settings including cancer care. This systematic review focussed on studies that were published from or used the instruments of CAHPS, CPES, and AOPSS and assessed factors influencing cancer patients’ experiences of care. We showed that cancer type, prognosis, and demographic characteristics have now been consistently linked with variation in patients’ experiences. Understanding how experiences vary between different populations and what actually lies beneath these differences, can help practitioners and policy makers prioritise initiatives to improve patients’ experiences within their cancer care systems. These findings show that improvement efforts in cancer care experience are not yet meeting the needs of all groups in society and that ideal cancer care pathways cannot therefore be designed as “one size fits all” approach. Our findings underline the importance of actively designing patient or person-centred systems around patients’ particular characteristics, their differing cancers, and the different trajectories they take through their care journeys.

## Contributors

SA, and ED designed the study, and decided the analytic approach, and agreed on eligible articles. MA assessed the quality of the articles. All authors (SA, MA, JR, MB, and ED) contributed to the interpretation of the results and the writing of this manuscript.

### Funding

This research received no specific grant from any funding agency in the public, commercial or not-for-profit sectors. This work is part of Saleh Alessy's PhD project, which is fully sponsored by Ministry of Education, Riyadh, Saudi Arabia.

### Data sharing statement

Data extraction tables and figures are available from SA.

## Declaration of interests

None.

## References

[1] Mollica M.A., Lines L.M., Halpern M.T. (2017). Patient experiences of cancer care: scoping review, future directions, and introduction of a new data resource: surveillance epidemiology and end results-consumer assessment of healthcare providers and systems (SEER-CAHPS). Patient Exp J.

[2] Evensen C.T., Yost K.J., Keller S. (2019). Development and testing of the CAHPS cancer care survey. J Oncol Pract.

[3] Iversen H.H., Holmboe O., Bjertnæs Ø.A. (2012). The cancer patient experiences questionnaire (CPEQ): reliability and construct validity following a national survey to assess hospital cancer care from the patient perspective. BMJ Open.

[4] Pinder R.J., Ferguson J., Møller H. (2016). Minority ethnicity patient satisfaction and experience: results of the national cancer patient experience survey in England. BMJ Open.

[5] Pham T.M., Gomez-Cano M., Salika T., Jardel D., Abel G.A., Lyratzopoulos G. (2019). Diagnostic route is associated with care satisfaction independently of tumour stage: evidence from linked English cancer patient experience survey and cancer registration data. Cancer Epidemiol.

[6] Gomez-Cano M., Lyratzopoulos G., Abel G.A. (2019). Patient experience drivers of overall satisfaction with care in cancer patients: evidence from responders to the English Cancer patient experience survey. J Patient Exp.

[7] Saunders C.L., Abel G.A., Lyratzopoulos G. (2014). What explains worse patient experience in London? Evidence from secondary analysis of the cancer patient experience survey. BMJ Open.

[8] Mollica M.A., Weaver K.E., McNeel T.S., Kent E.E. (2018). Examining urban and rural differences in perceived timeliness of care among cancer patients: a SEER-CAHPS study. Cancer.

[9] Lines L.M., Cohen J., Halpern M.T., Smith A.W., Kent E.E. (2019). Care experiences among dually enrolled older adults with cancer: SEER-CAHPS, 2005–2013. Cancer Causes Control.

[10] Bracher M., Corner D.J., Wagland R. (2016). Exploring experiences of cancer care in Wales: a thematic analysis of free-text responses to the 2013 Wales Cancer Patient Experience Survey (WCPES). BMJ Open.

[11] NHS Improvement. National Cancer Patient Experience Survey. NHS England. 2022. https://www.ncpes.co.uk/. Accessed 13 April 2022.

[12] Chawla N., Urato M., Ambs A. (2015). Unveiling SEER-CAHPS®: a new data resource for quality of care research. J Gen Intern Med.

[13] Chadder J., Zomer S., Lockwood G. (2018). Understanding the experiences of cancer patients as they transition from treatment to primary and community care: a Pan-Canadian study of over 13,000 cancer survivors. J Glob Oncol Am Soc Clin Oncol.

[14] Bridge E., Gotlib Conn L., Dhanju S., Singh S., Moody L. (2019). The patient experience of ambulatory cancer treatment: a descriptive study. Curr Oncol.

[15] Doyle C., Lennox L., Bell D. (2013). A systematic review of evidence on the links between patient experience and clinical safety and effectiveness. BMJ Open.

[16] Alessy S.A., Lüchtenborg M., Davies E.A. (2019). How have patients’ experiences of cancer care been linked to survival? A systematic review. Patient Exp J.

[17] Liberati A., Altman D.G., Tetzlaff J. (2009). The PRISMA statement for reporting systematic reviews and meta-analyses of studies that evaluate health care interventions: explanation and elaboration. PLoS Med.

[18] National Institutes of Health (2021). https://www.nhlbi.nih.gov/health-topics/study-quality-assessment-tools.

[19] Lyratzopoulos G., Neal R.D., Barbiere J.M., Rubin G.P., Abel G.A. (2012). Variation in number of general practitioner consultations before hospital referral for cancer: findings from the 2010 national cancer patient experience survey in England. Lancet Oncol.

[20] Bone A., Mc Grath-Lone L., Day S., Ward H. (2014). Inequalities in the care experiences of patients with cancer: analysis of data from the national cancer patient experience survey 2011–2012. BMJ Open.

[21] El Turabi A., Abel G.A., Roland M., Lyratzopoulos G. (2013). Variation in reported experience of involvement in cancer treatment decision making: evidence from the national cancer patient experience survey. Br J Cancer.

[22] Saunders C.L., Abel G.A., Lyratzopoulos G. (2015). Inequalities in reported cancer patient experience by socio-demographic characteristic and cancer site: evidence from respondents to the English cancer patient experience survey. Eur J Cancer Care (Engl).

[23] Stephens R., Morris C., West R. (2015). Keeping the customer satisfied #2 It Is OK To Ask - who are we asking, and who participates? Further findings from the national cancer patient experience survey 2013. Eur J Cancer Care (Engl).

[24] Mc Grath-Lone L., Day S., Schoenborn C., Ward H. (2015). Exploring research participation among cancer patients: analysis of a national survey and an in-depth interview study. BMC Cancer.

[25] Trenchard L., McGrath-Lone L., Ward H. (2014). Ethnic variation in patients’ ratings of communication: analysis of national cancer patient experience survey data. Lancet.

[26] Cunningham M., Wells M. (2017). Qualitative analysis of 6961 free-text comments from the first national cancer patient experience survey in Scotland. BMJ Open.

[27] Hulbert-Williams N.J., Plumpton C.O., Flowers P. (2017). The cancer care experiences of gay, lesbian and bisexual patients: a secondary analysis of data from the UK Cancer Patient Experience Survey. Eur J Cancer Care (Engl).

[28] Nartey Y., Tata L.J., Khakwani A. (2022). Using patient experiences to evaluate care and expectations in lung cancer: analysis of the English cancer patient experience survey linked with the national cancer registry. Support Care Cancer.

[29] Trenchard L., Mc Grath-Lone L., Ward H. (2016). Ethnic variation in cancer patients’ ratings of information provision, communication and overall care. Ethn Health.

[30] Shirk J.D., Tan H.J., Hu J.C., Saigal C.S., Litwin M.S. (2016). Patient experience and quality of urologic cancer surgery in US hospitals. Cancer.

[31] Halpern M.T., Urato M.P., Kent E.E. (2017). The health care experience of patients with cancer during the last year of life: analysis of the SEER-CAHPS data set. Cancer.

[32] Chan E., David E.A., Eguchi M., Cockburn M., Farias A.J. (2020). Clinical Research (Excluding Clinical Trials).

[33] Loiselle C.G. (2019). Cancer information-seeking preferences linked to distinct patient experiences and differential satisfaction with cancer care. Patient Educ Couns.

[34] Mollica M.A., Buckenmaier S.S., Halpern M.T. (2020). Perceptions of care coordination among older adult cancer survivors: a SEER-CAHPS study. J Geriatr Oncol.

[35] Halpern M.T., Urato M.P., Lines L.M., Cohen J.B., Arora N.K., Kent E.E. (2018). Healthcare experience among older cancer survivors: analysis of the SEER-CAHPS dataset. J Geriatr Oncol.

[36] Lines L.M., Barch D.H., Zabala D., Halpern M.T., Jacobsen P., Mollica M. (2020). Associations between perceptions of care experiences and receipt of mental health care among older adults with cancer. J Clin Oncol.

[37] Lines L., Kirschner J., Halpern M., Kent E., Mollica M., Smith A. (2020). Proceedings of the APHA's VIRTUAL Annual Meeting and Expo (Oct. 24-28).

[38] Fauer A, Choi SW, Wallner LP, Davis MA, Friese CR. Understanding quality and equity : patient experiences with care in older adults diagnosed with hematologic malignancies. Cancer Causes Control 2021. 10.1007/s10552-021-01395-4. PMC7946754 33566250

[39] Coronado A.C., Tran K., Chadder J. (2017). The experience of patients with cancer during diagnosis and treatment planning: a descriptive study of Canadian survey results. Curr Oncol.

[40] Salika T., Abel G.A., Mendonca S.C. (2018). Associations between diagnostic pathways and care experience in colorectal cancer: evidence from patient-reported data. Frontline Gastroenterol.

[41] Farias A.J, Toledo G, Ochoa C.Y, Hamilton A.S. Racial /ethnic disparities in patient experiences with health care in association with earlier stage at colorectal cancer diagnosis findings from the SEER-CAHPS data. 2021: 1–9. 10.1097/MLR.0000000000001514 33528232

[42] Farias A.J., Ochoa C.Y., Toledo G., In S., Ann B., Xianglin S.H. (2020). Racial /ethnic differences in patient experiences with health care in association with earlier stage at breast cancer diagnosis : findings from the SEER ‑ CAHPS data. Cancer Causes Control.

[43] Navarro S., Hu X., Mejia A. (2022). Racial/ethnic disparities in patient experiences with care and Gleason score at diagnosis of prostate cancer: a SEER-CAHPS study. Cancer Causes Control.

[44] Richard S.C., Shaw C.E. (2016). How do myeloma patients’ experiences before and during diagnosis compare to those for all cancers? Findings from secondary analysis of the National Cancer Patient Experience Survey 2014. Br. J. Haematol.

[45] Lacey K., Bishop J.F., Cross H.L., Chondros P., Lyratzopoulos G., Emery J.D. (2016). Presentations to general practice before a cancer diagnosis in Victoria: a cross-sectional survey. Med J Aust.

[46] Wagland R., Bracher M., Drosdowsky A. (2017). Differences in experiences of care between patients diagnosed with metastatic cancer of known and unknown primaries: mixed-method findings from the 2013 cancer patient experience survey in England. BMJ Open.

[47] Abel G.A., Saunders C.L., Lyratzopoulos G. (2016). Post-sampling mortality and non-response patterns in the English cancer patient experience survey: implications for epidemiological studies based on surveys of cancer patients. Cancer Epidemiol.

[48] Alessy S.A., Davies E.A., Rawlinson J., Baker M., Lüchtenborg M. (2019). How representative are colorectal, lung, breast and prostate cancer patients responding to the national cancer patient experience survey (CPES) of the cancer registry population in England? A population-based case control study. BMJ Open.

[49] Nartey Y., Stewart I., Khakwani A. (2020). Is the English cancer patient experience survey representative? A comparative analysis with the national lung cancer audit. Lung Cancer.

[50] Pham T.M., Abel G.A., Gomez-Cano M., Lyratzopoulos G. (2019). Predictors of postal or online response mode and associations with patient experience and satisfaction in the English cancer patient experience survey. J Med Internet Res.

[51] Dalby M., Hill A., Nabhani-Gebara S. (2021). Analysis of local qualitative cancer patient experience alongside the 2019 results of the UK national cancer patient experience survey. Int J Pharm Pract.

[52] Griffiths P., Simon M., Richardson A., Corner J. (2013). Is a larger specialistnurse workforce in cancer care associated with better patient experience? Cross-sectional study. J Health Serv Res Policy.

[53] Clucas C. (2016). Cancer patients’ respect experiences in relation to perceived communication behaviours from hospital staff: analysis of the 2012–2013 national cancer patient experience survey. Support Care Cancer.

[54] Mendonca S.C., Abel G.A., Saunders C.L., Wardle J., Lyratzopoulos G. (2016). Pre-referral general practitioner consultations and subsequent experience of cancer care: evidence from the English cancer patient experience survey. Eur J Cancer Care (Engl).

[55] Saunders C., Elliott M.N., Lyratzopoulos G., Abel G.A. (2016). Do differential response rates to patient surveys between organizations lead to unfair performance comparisons?. Med Care.

[56] Abel G.A., Mendonca S.C., McPhail S., Zhou Y., Elliss-Brookes L., Lyratzopoulos G. (2017). Emergency diagnosis of cancer and previous general practice consultations: insights from linked patient survey data. Br J Gen Pract.

[57] Mollica M.A., Enewold L.R., Lines L.M. (2017). Examining colorectal cancer survivors’ surveillance patterns and experiences of care: a SEER-CAHPS study. Cancer Causes Control.

[58] Mollica M., Lines L.M., McNeel T.S. (2019). Patient experiences of care in localized prostate cancer. J Clin Oncol.

[59] Singer E.S., Merritt R.E., D'Souza D.M., Moffatt-Bruce S.D., Kneuertz P.J. (2019). Patient satisfaction after lung cancer surgery: do clinical outcomes affect hospital consumer assessment of health care providers and systems scores?. Ann Thorac Surg.

[60] Fitch M.I., Coronado A.C., Schippke J.C., Chadder J., Green E. (2019). Exploring the perspectives of patients about their care experience: identifying what patients perceive are important qualities in cancer care. Support Care Cancer.

[61] Fauer A., Wallner L.P., Davis M.A., Choi S.W., Friese C.R. (2021). Health care experiences for older adults diagnosed with leukemia and lymphoma: factors associated with emergency department use, timeliness and access of health care. J Geriatr Oncol.

[62] Halpern M.T., Cohen J., Lines L.M., Mollica M.A., Kent E.E. (2020). Associations between shared care and patient experiences among older cancer survivors. J Cancer Surviv.

[63] Snyder R.A., Wardrop R., McLain A.C., Parikh A.A., Cass A.L. (2021). Association of patient experience with guideline-concordant colon cancer treatment in the elderly. JCO Oncol Pract.

[64] Alessy S.A., Lüchtenborg M., Rawlinson J., Baker M., Davies E.A. (2021). Being assigned a clinical nurse specialist is associated with better experiences of cancer care: English population-based study using the linked national cancer patient experience survey and cancer registration dataset. Eur J Cancer Care (Engl).

[65] Prue G., O'Connor D., Brown M., Santin O. (2021). Exploring patient experiences of cancer care in Northern Ireland: a thematic analysis of free-text responses to the 2018 Northern Ireland patient experience survey (NICPES). BMC Health Serv Res.

[66] Watson L., Qi S., Photitai E., Deiure A. (2021). A cross-sectional analysis of ambulatory oncology experience by treatment intent. Curr Oncol.

[67] Hamad J., Mohan C., Rotter J.S. (2020). Time-to-treatment after cancer diagnosis and the patient experience with care: a SEER-CAHPS study. J Am Coll Surg.

[68] Institute of Medicine (2013).

[69] Wolf J.A. (2018). The consumer has spoken: patient experience is now healthcare's core differentiator. Patient Exp J.

[70] Saunders C., Carter D.J., Jordan A., Duffield C., Bichel-Findlay J. (2016). Cancer patient experience measures: an evidence review. J Psychosoc Oncol.

[71] Care Quality Commission (2021). https://www.cqc.org.uk/publications/surveys/adult-inpatient-survey-2020.

[72] Alessy S., Lüchtenborg M., Davies E.A. (2019). Comparison of the linked cancer registry and cancer patient experience survey datasets in england and the United States. Abstr Br Med J Publ Group.

[73] Abel G.A., Gomez-Cano M., Pham T.M., Lyratzopoulos G. (2019). Reliability of hospital scores for the Cancer Patient Experience Survey: analysis of publicly reported patient survey data. BMJ Open.

[74] Gomez-Cano M., Lyratzopoulos G., Campbell J.L., Elliott M.N., Abel G.A. (2022). The underlying structure of the English Cancer Patient Experience Survey: factor analysis to support survey reporting and design. Cancer Med.

[75] Royce T.J., Sanoff H.K., Rewari A. (2020). Telemedicine for Cancer Care in the Time of COVID-19. JAMA Oncol.

[76] Richards T. (2021). Can you do what I'm asking you to do?. BMJ.

